# pH- and Metal Ion- Sensitive Hydrogels based on *N*-[2-(dimethylaminoethyl)acrylamide]

**DOI:** 10.3390/polym8060233

**Published:** 2016-06-15

**Authors:** Leena Nebhani, Veena Choudhary, Hans-Jürgen P. Adler, Dirk Kuckling

**Affiliations:** 1 Centre for Polymer Science and Engineering, Indian Institute of Technology, Hauz Khas, New Delhi 110016, India; veenach@hotmail.com; 2 Institute for Macromolecular Chemistry and Textile Chemistry, Dresden University of Technology, D-01062 Dresden, Germany; hansjuergen_adler@yahoo.de; 3 Chemistry Department, University of Paderborn, Warburger Str. 100, D-33098 Paderborn, Germany

**Keywords:** smart hydrogels, pH sensitive, metal ion sensitive, *N*-[2-(dimethylaminoethyl)acrylamide], photopolymerization

## Abstract

Smart hydrogels are promising materials for actuators and sensors, as they can respond to small changes in their environment with a large property change. Hydrogels can respond to a variety of stimuli, for example temperature, pH, metal ions, *etc*. In this article, the synthesis and characterization of polyampholyte hydrogels based on open chain ligands showing pH and metal ion sensitivity are described. Copolymer and terpolymer gels using different mixtures of monomers *i.e*., *N*-[2-(dimethylaminoethyl)acrylamide] (DMAEAAm), *N,N*-dimethylacrylamide (DMAAm), acrylic acid (AA) and 2-acrylamido-2-methyl-1-propanesulphonic acid (AMPS), have been synthesized. The effect of copolymer composition, *i.e*., the ratio and amount of ionic monomers and the degree of crosslinking on the swelling characteristics, was evaluated as a function of pH. On this basis, metal ion sensitivity measurements were performed at selected pH values. The metal ion sensitivity was measured by varying the concentration of Cu^2+^, Zn^2+^ and Ag^+^ ions under acidic pH conditions.

## 1. Introduction

Microsystems based on hydrogels are widely used as sensors and actuators [1,2,3,4]. pH-responsive hydrogels [5,6,7,8,9] can be used, for example, in micromechanical cantilevers [10], microvalves [11] and microfluidic devices [12]. Hydrogels can undergo phase transitions due to various stimuli, for example pH, temperature, metal ions, *etc*. [13,14,15]. Phase transitions due to temperature [16] and pH [17,18] are widely reported; however, a limited number of studies have been carried out on phase transition due to metal ions. The phase transition in hydrogels due to metal ions can make hydrogels very important materials for various environmental and clinical applications. There is a great need for sensors based on hydrogels that can detect metal ions in aqueous solutions [19,20]. In order to make hydrogels sensitive to metal ions, it is necessary to attach groups or ligands onto the hydrogel network, which can selectively bind with metal ions. The approach, which had been earlier used for the preparation of metal ion-sensitive hydrogels, incorporates crown ether [21] or other cyclic ligands into the hydrogel network [22]. The crown ether selectively binds to the metal ions. This binding of metal ions brings free charges into the gel network, which consequently causes hydration and, thus, swelling. Sensors of this kind have been reported for the detection of Na^+^ [23], Pb^2+^ [24,25], Ba^2+^ [25] and K^+^ [23,25]. However, for these cyclic ligands to be incorporated as a pendant group into sensitive polymers, they have to be functionalized first. This functionalization of the cyclic ligand is a multi-step process involving various steps of protection and deprotection. Another limiting factor of cyclic ligands is their cost. These cyclic ligands are mostly studied with temperature-sensitive polymers and, therefore, have a limited temperature range, in which the sensitivity toward the metal ions can be controlled.

In this work, the synthesis and characterization of smart hydrogels based on *N*-[2-(dimethylaminoethyl)acrylamide] (DMAEAAm) possessing pH and metal ion sensitivity is reported. The approach used in this work makes use of open chain ligands in combination with a hydrophilic monomer *N,N*-dimethylacrylamide (DMAAm) to maintain swellability over a wide range of pH. The monomer selected for the study is *N*-[2-(dimethylaminoethyl)acrylamide] (DMAEAAm) in which dimethylamino groups are the potential sites that are ionizable, as well as being able to have interaction with metal ions. Other ionizable monomers, for example, acrylic acid (AA) and 2-acrylamido-2-methyl-1-propanesulphonic acid (AMPS), were also used to modulate the swelling properties of the resulting hydrogels by competing ionic interactions. Depending on the monomer combination used, three different systems were prepared.

System I A two-component system consisting of *N*-[2-(dimethylaminoethyl)acrylamide] (DMAEAAm) and *N,N*-dimethylacrylamide (DMAAm).

System II A three-component system consisting of *N*-[2-(dimethylaminoethyl) acrylamide] (DMAEAAm), *N,N*-dimethylacrylamide (DMAAm) and acrylic acid (AA).

System III A three-component system consisting of *N*-[2-(dimethylaminoethyl) acrylamide] (DMAEAAm), *N,N*-dimethylacrylamide (DMAAm) and 2-acrylamido-2-methyl-1-propane sulfonic acid (AMPS).

In all three systems used for the preparation of hydrogels, *N,N^′^*-methylenebis(acrylamide) (BIS) and Irgacure-2959 (1-[4-(2-hydroxyethoxy)-phenyl]-2-hydroxy-2-methyl-1-propane-1-one) were used as a crosslinking agent and a photo initiator, respectively.

## 2. Materials and Methods

2,6-Di-tert-butyl-4-methyl phenol, 2-acrylamido-2-methyl-1-propanesulphonic acid [AMPS] (Aldrich), 2-hydroxy-1-[4-(2-hydroxyethoxy)phenyl]-2-methyl-1-propanone [Irgacure 2959] (Aldrich), acryloyl chloride (Fluka, St. Louis, MO, USA), 0.2 N sodium hydroxide (Aldrich, St. Louis, MO, USA), anhydrous sodium carbonate, anhydrous sodium sulfate, copper slfate anhydrous (Merck, Darmstadt, Germany), *N*,*N*-dimethylethylenediamine (Fluka), zinc sulfate hepta hydrate, silver nitrate, silica gel, ethyl acetate, methanol and triethylamine were used as received. Phosphate-buffered saline was prepared by dissolving one tablet in 200 mL double-distilled water providing a pH of 7.5. Dichloromethane was distilled over CaCl_2_. Acrylic acid (AA) (Fluka) and *N,N*-dimethylacrylamide (DMAAm) (Fluka) were distilled under vacuum and stored at a low temperature. *N*-[2-(Dimethylaminoethyl)acrylamide] (DMAEAAm) monomer was synthesized according to the procedure discussed below [26].

In a three-necked flask equipped with a condenser, a thermometer, an addition funnel and a stirrer are placed dichloromethane (100 mL), *N,N*-dimethylethylenediamine (0.1 mol), anhydrous sodium carbonate (0.1 mol) and 2,4-di-tert-butyl-4-methyl phenol (0.2% with respect to acryloyl chloride) and then cooled to 0–5 °C. Acryloyl chloride (0.1 mol) was slowly dropped to the mixture under nitrogen. After the addition of acryloyl chloride, the reaction mixture was allowed to warm up to room temperature and stirred for 4 h. The resulting solution was centrifuged to remove sodium carbonate, dried with sodium sulfate and evaporated. The product was purified by column chromatography eluting with ethyl acetate:methanol (3:1) (*v/v*) gradually increasing to 1:1 under the pressure of nitrogen to give a light yellowish oil. TLC of the product was checked in methanol:triethylamine (20:1). The *R*_f_ value of the product was found to be 0.58. The product was characterized by ^1^H-NMR and ^13^C-NMR. The crude yield was 10.0 g (70%), and the yield after flash chromatography was 9.0 g (63%).

^1^H NMR (CDCl_3_) δ (ppm) = 6.6 (broad singlet, NH), 5.5–6.2 (multiplet, 3H, CH_2_=CH), 3.35 (quartet like multiplet, 2H, NHCH_2_), 2.4 (triplet, 2H, CH_2_N), 2.2 (singlet, 6H, N(CH_3_)_2_).

^13^C NMR (CDCl_3_) δ (ppm) = 165.45 (C=O), 130.74 (=CH), 125.73 (CH_2_=), 57.51 (NHCH_2_), 44.84 (CH_3_), 36.56 (CH_2_N).

**Scheme 1:**
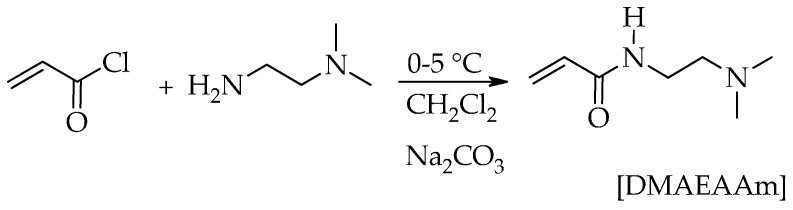
Synthesis of monomer *N*-[2-(dimethylaminoethyl)acrylamide].

The reaction scheme for the synthesis of *N*-[2-(dimethylaminoethyl)acrylamide] (DMAEAAm) is shown in Scheme 1.

### 2.1. Synthesis of Hydrogels

**Scheme 2:**
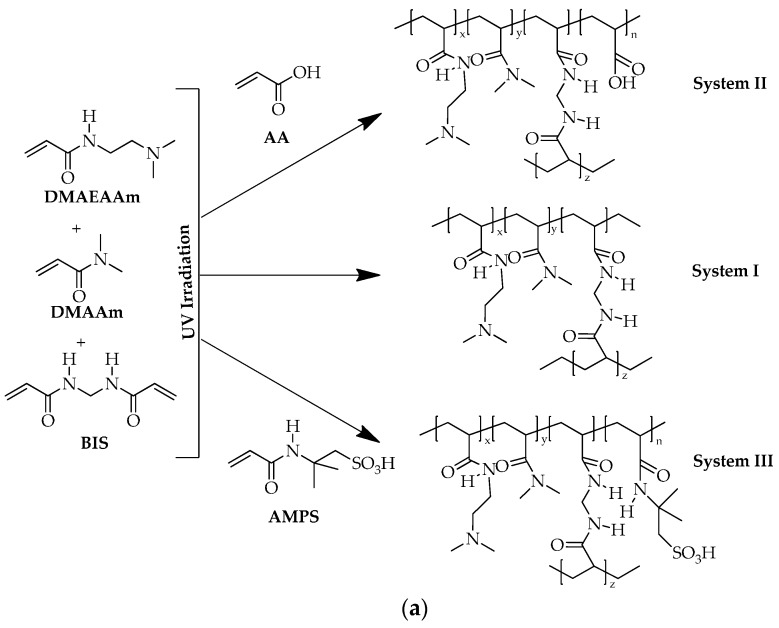

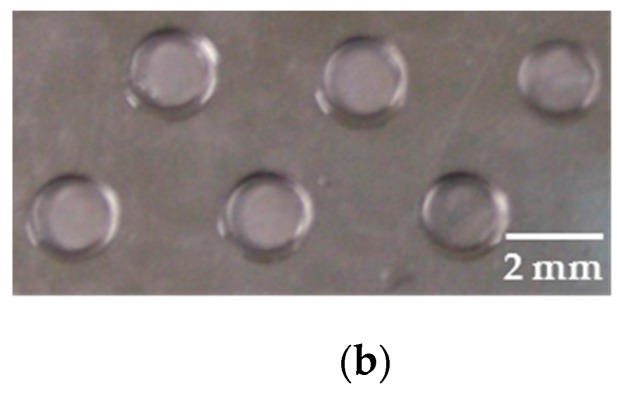
(**a**) Copolymerization of DMAEAAm and DMAAm (System I), DMAEAAm, DMAAm and AA (System II) and DMAEAAm, DMAAm and AMPS (System III); (**b**) shape of hydrogels after photo polymerization.

A set-up consisting of a UV lamp (100 W Hg lamp, Osram, Munich, Germany) equipped with an optical lens, a mirror and connected to a water circulating thermostat (HAAKE) operating at a temperature of 7.6 °C was used for the photo polymerization process. No special filters were used during photo polymerization. Glass slides separated by a spacer (thickness of 500 µm) were used as the reactor for polymerization, allowing UV radiation above 310 nm to pass through it. The UV lamp provides the radiation in the range from 265–360 nm. Hydrogels were prepared by photo polymerization of the specific composition of monomer solutions at room temperature. All of the solutions were degassed with argon prior to the polymerization. Three different copolymerization systems were prepared with the aim to investigate systematically the effect of crosslinking density and the concentration of ionic monomers. For photo patterning, the glass reactor was filled with the argon purged solution of the monomers, crosslinking agent, photo initiator and water. The reactor was covered with a glass plate followed by a mask having a pattern of a circular disc (diameter of 2 mm). The photo polymerization time required to produce a pattern varies from 20–50 s for the different systems. After irradiating for the desired time intervals, the patterned hydrogels were maintained in distilled water for five days with a change of water each day for the removal of unreacted starting materials from the samples. Scheme 2 shows the different systems of hydrogels studied. The compositional details of each system are summarized in Table 1.

### 2.2. Characterization of Hydrogels

The diameter of the gels was measured by a stereomicroscope (Hund, Wetzlar, Germany) equipped with a digital camera (JVC) having a charge coupled device (CCD, which is an image sensor, consisting of an integrated circuit containing an array of linked, or coupled, light-sensitive capacitors). The swelling ratio for a bulk gel was determined by taking the ratio of the diameter of the swollen hydrogel and the diameter of the gel in the reference state (e.g., as prepared). The degree of swelling is expressed as the ratio of the gel volume in equilibrium state (*V*) to the volume at preparation (*V*_0_), calculated by equation shown below:

*V*/*V*_0_ = (*d*/*d*_0_)^3^ (1)

*d* is the diameter of the swollen hydrogel, and *d*_0_ is the diameter of the hydrogel in the reference state.

For pH sensitivity measurements, swollen hydrogels of a known diameter were immersed in a medium of known pH, and the diameter was determined by the microscope. For one measurement, the average diameter of three dots was taken for the calculation of the volume degree of swelling. Before each measurement, the hydrogels were equilibrated for 2 h in that pH medium. Buffer solutions having pH ranging from 2–12 were prepared by dissolving Component A (composition in 1 liter double-distilled water): 6.008 g citric acid monohydrate (35%), 1.769 g boric acid (10.4%), 3.893 g potassium hydrogen sulfate (23.0%), 5.266 g 5,5-diethylbarbituric acid (31.1%); Component B: 0.2 N sodium hydroxide solution. For the determination of metal ion sensitivity, 0.01 M solutions of CuSO_4_, ZnSO_4_ and AgNO_3_ were prepared. Hydrogel samples were immersed in 0.01 M acetic acid, and the change in diameter of hydrogels after the addition of 10 µL of metal ion solution at equilibrium, *i.e*., 2 h was determined.

## 3. Results and Discussion

### 3.1. pH Sensitivity

#### 3.1.1. Effect of Crosslinking

**Figure 1:**
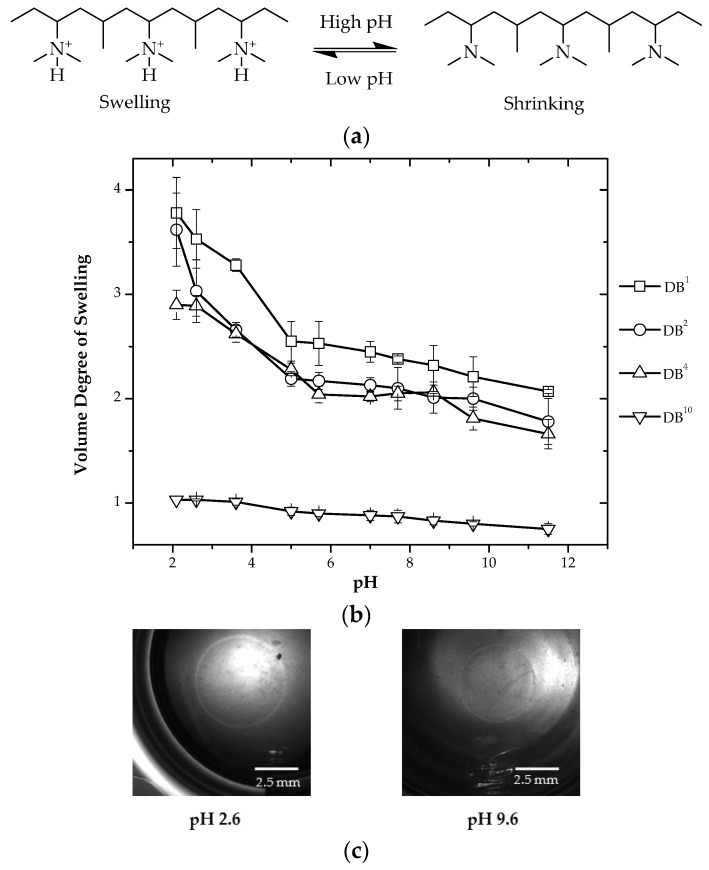
(**a**) Chemical composition of System I hydrogels in different pH states; (**b**) variation of the volume degree of swelling with pH for different compositions of hydrogels; (**c**) optical micrographs showing the change in diameter of DB^4^ hydrogels at pH 2.6 and 9.6.

In this study, System I is a two-component system having DMAEAAm and DMAAm. The effect of crosslinker concentration on the swelling behavior of hydrogels under different pH conditions is shown in Figure 1.

The system having the lowest amount of crosslinking (1 mol %) shows the highest swelling, and the system having the highest amount of crosslinking (10 mol %) shows the lowest swelling. There is an increase in the volume degree of swelling with a change of pH to a lower value. At higher pH values, the non-ionized pendant dimethylamino groups of the DMAEAAm moiety are less hydrophilic. As a consequence, they likely associate and exclude water from the polymer network (the collapsed state). When the solution pH is decreased to values below the p*K*_a_ of the ionizable dimethylamino group, protonation occurs, resulting in the formation of positively-charged pendant groups in the polymeric backbone. Protonation of pendant dimethylamino groups gives rise to two phenomena, each of which contributes to the swelling behavior of the hydrogel. First, the positively-charged amine groups repel one another, causing the polymer chains to move spatially. In addition, to maintain the electro neutrality (Donnan equilibrium) within the gel, solvated counter ions are drawn into the polymer structure; thus, the positively-charged amine groups become caged by these solvated counter ions and additional water molecules. Both of these phenomena lead to the increased sorption of water (degree of hydration) into the hydrogel, causing it to swell, as shown in Figure 1.

#### 3.1.2. Effect of Ionizable Monomer

In Systems II and III, the effect of ionizable monomer at different pH was investigated. In the systems that are both crosslinked, as well containing acidic (anionic) and basic (cationic) pendant groups, swelling and deswelling are dependent on solution pH, as well the ionic composition of the hydrogel. In the case of hydrogels containing acidic pendant groups, the volume degree of swelling increases as the pH of the solution increases, whereas a decrease in the pH enhances the swelling of hydrogels containing basic pendant groups. In case of amphoteric gels, the volume degree of swelling is also dependent on the relative amount of ionic groups. Therefore, specific bonding between the anionic and cationic groups should be considered. The specific bonding between cationic and anionic groups can act as a cross-linking structure, which can result in a decrease in volume degree of swelling.

**Figure 2:**
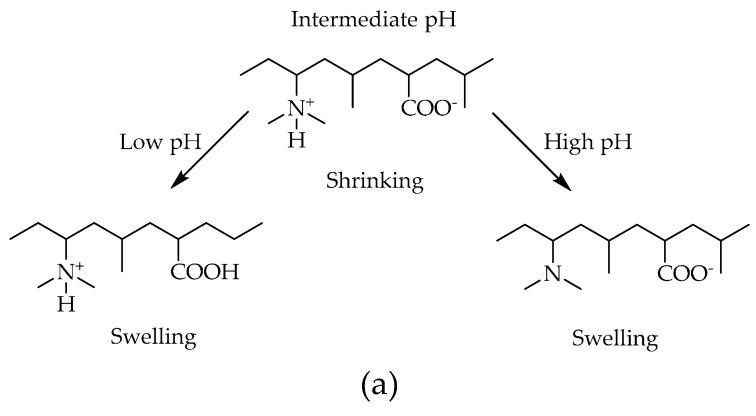

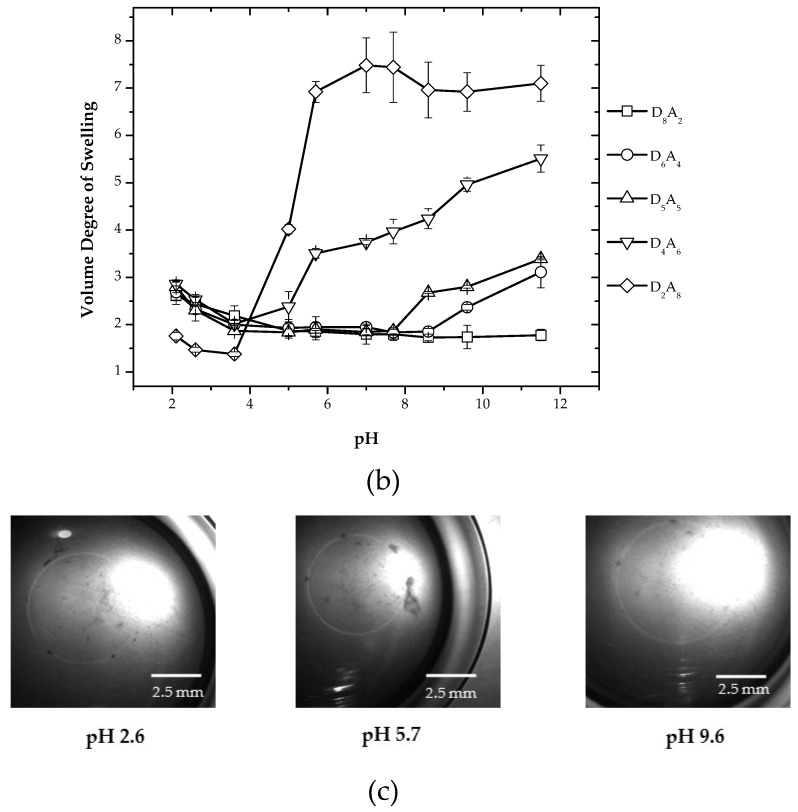
(**a**) Chemical composition of System II hydrogels in different pH states; (**b**) variation of the volume degree of swelling for different compositions of hydrogels; (**c**) optical micrographs showing the change in diameter of D_5_A_5_ hydrogels at pH 2.6, 5.7 and 9.6.

System II is the three-component system having DMAEAAm, AA and DMAAm, from which DMAEAAm is the basic component and AA is the acidic component. The composition was chosen in such way that a DMAAm content of 50% (with respect to non-crosslinkable monomers) was maintained. The hydrogels were synthesized by varying the ratio of two ionizable components, which are in an ionized state at different pH conditions. Therefore, on one extreme, the system has an excess of basic groups, and on the other extreme, the system has an excess of acidic groups; and in between these extreme compositions is the system having an equal ratio of basic and acidic groups. Depending on the state of the particular co-monomer, different types of interactions are present inside the hydrogel network. The variation of the volume degree of swelling with respect to pH for hydrogels incorporating a weakly-ionizable component added to the system of DMAEAAm and DMAAm having a fixed concentration (2 mol %) of BIS is shown in Figure 2.

The weakly-ionizable components in System II are both DMAEAAm and AA. The concentration of AA is varied from 10 to 40% with respect to the total monomer content. Figure 2 shows different compositions of hydrogels for System II under varying pH conditions. The hydrogels having the composition of D8A2, *i.e*., having the lowest amount of AA and the highest amount of DMAEAAm, shows no swelling at a pH ranging from 7–12, while swelling is stronger in the pH range of less than five, which can be explained due to the protonation of the dimethylamino group of DMAEAAm. For composition D6A4, swelling was observed in a pH range greater than nine and less than five. This can be explained by the ionization of the carboxylic groups of AA at a higher pH range and the protonation of the dimethylamino groups of DMAEAAm in the low pH range. In the intermediate pH range (5–9), where both carboxylic and dimethylamino groups are ionized, the volume degree of swelling is lowest due to the ionic interactions between positive charges of the dimethylamino group and negative charges of the carboxylic groups. This intermediate pH range is generally referred to as the isoelectric pH range (pI), where the electrostatic attraction due to opposite charges is highest. Therefore, in the pI range, there are two types of crosslinks, one due to the BIS and the other due to the creation of electrostatic junction points (ionic interactions).

For composition D_5_A_5_, swelling is observed in a pH range greater than 7.7 and less than 3.6, and in between pH 3.6 and 7.7, there is very less swelling. For composition D_4_A_6_, higher swelling was observed in the pH range greater than 3.6 and less than pH 3.6; at pH 3.6, there is a minimum amount of swelling. As the ratio of ionizable components is varied from 1:1, the isoelectric pH range decreases because of the imbalance of the ratio of positive and negative charges. For composition D_2_A_8_, a higher amount of swelling was observed at pH greater than 3.6, and a relatively small amount of swelling is observed at a pH lower than 3.6; this is because less protonated dimethylamino groups from DMAEAAm are available. The variation of the volume degree of swelling depending on the composition of hydrogels and varying pH can be explained by the different states of ionization of two ionizable co-monomers. At higher pH values, the pendant dimethylamino groups of DMAEAAm present in the non-ionized state are less hydrophilic. However, carboxylic groups present in AA are in the ionized state at higher pH values. This ionization is reversed when the pH is decreased; dimethylamino groups are in the ionized state at low pH, while carboxylic groups are in the non-ionized state in low pH medium. Therefore, for System II, hydrogels are in the swollen state at both high and low pH.

System III is also a three-component system having DMAEAAm, AMPS and DMAAm, in which DMAEAAm is the basic component and AMPS the acidic component. The hydrogels were synthesized by varying the ratio of two ionizable components, from which AMPS is in the ionized state over the whole pH range.

**Figure 3:**
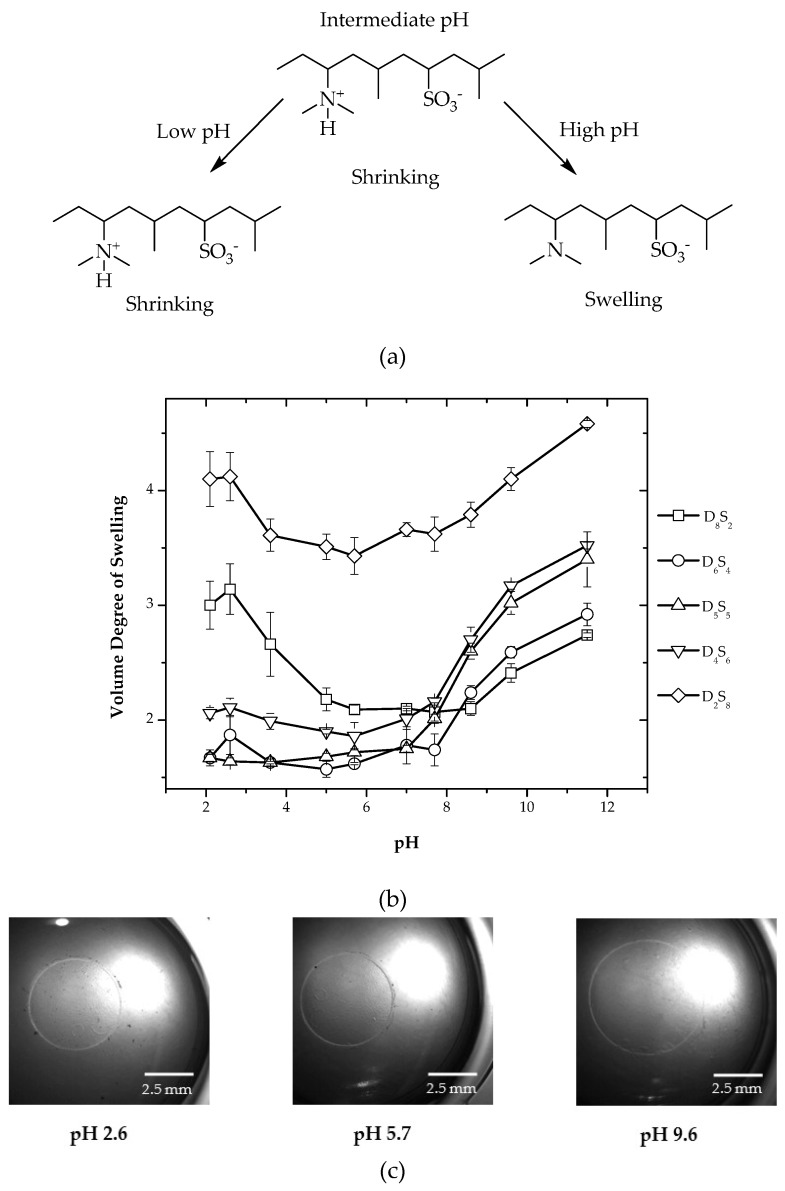
(**a**) Chemical composition of System III hydrogels in different pH states; (**b**) variation of the volume degree of swelling for different compositions of hydrogels; (**c**) optical micrograph showing the change in the diameter of D_5_S_5_ hydrogels at pH 2.6, 5.7 and 9.6.

AMPS has a strongly ionizable sulfonate group; it remains in the dissociated state in the pH range of 2–12, and therefore, hydrogels derived from AMPS exhibit a pH-independent swelling behavior. The effect of the addition of a second ionizable component, which is completely ionized over the whole pH range, is shown in Figure 3. The concentration of AMPS was varied from 10%–40% with respect to total monomer content.

For composition D_8_S_2_, having the lowest amount of AMPS and the highest amount of DMAEAAm, it showed higher swelling in a pH range higher than nine and a pH lower than six, which is due to the protonation of the dimethylamino group of DMAEAAm in the lower pH region. At higher pH, dimethylamino groups are deprotonated, and therefore, higher swelling as compared to hydrogels having only DMAEAAm as the ionizable component is due to the sulfonate groups of AMPS, which are ionized over the whole pH range. In the pH range of 6–9, there is a very small amount of swelling, which is due to the formation of additional ionic interaction between the positive charges of the protonated dimethylamino groups and the negative charge of the ionized sulfonic acid groups. For compositions around the isoelectric range (D_6_S_4_, D_5_S_5_ and D_4_S_6_), swelling was observed at a pH range > 7, and at a pH < 7, a very small amount of swelling was seen. This is because of the much lesser amount of ionic content, as positive charges due to protonated dimethylamino groups of DMAEAAm are electrostatically neutralized with negative charges due to the sulfonic acid groups of AMPS. For composition D_2_S_8_, a high amount of swelling is observed over the whole pH range due to the excess of sulfonate groups. The optical micrographs for composition D_5_S_5_ in three different pH conditions is shown in Figure 3.

System I is a weak basic polyelectrolyte gel with its classical swelling behavior. With the increase in pH, more and more side groups are deprotonated, finally resulting in a drastic decrease in swelling. For this system the critical pH value is around pH 5. The increase of the content of crosslinker screens this effect. Hence, the amount of crosslinker should be kept as low as possible, but not effecting the mechanical integrity. Systems II and III are polyampholyte gels with balanced and imbalanced charges. In addition to the polybase part, System II has a pH-dependent anionic component, whereas System III has an additional pH-independent component. Polyampholyte gels are of interest, since they show a unique behavior, e.g., increased swelling with increased ionic strength [27] or enhanced mechanical stability [28]. In order to minimize the effect of ionic strength, in this study, a particular buffer was used maintaining almost the same ionic strength over the whole pH range. Polyampholyte gels are obtained by copolymerizing a monomer with anionic functional groups and a monomer with cationic functional groups forming anionic–cationic charges in different polymer repeat units or polymerizing a zwitterionic monomer leading to the formation of anionic–cationic charges in the same polymer repeat units. However, the swelling capacity is larger for the first case [27]. Changes in both charge density and crosslink density are suggested to contribute to the swelling mechanism of polyampholytic hydrogels. It has been shown that the swelling response of anionic offset polyampholytic and poly-zwitterionic hydrogels can be regarded as a super-position of responses measured for charge balanced and pure anionic hydrogels. This approximation was not valid for cationic offset hydrogels [27]. The charges within polyampholyte hydrogels can act as reversible crosslinks that also result in the decrease of the effective hydrogel charge density. The breakage of the physical crosslinks leads to the decrease of the effective crosslink density in the polymer network. For chargeable units within the polyampholyte gels, the swelling behavior is associated with the occurrence of an isoelectric point [29]. For gels with a significant charge imbalance, this behavior vanishes. It also can be seen that in System III, the anionic component is charged over the whole pH range, resulting in a stronger interaction with the cationic component and, hence, in a decreased swelling.

### 3.2. Metal Ion Sensitivity

#### 3.2.1. Effect of Crosslinking

In System I, the effect of crosslinking density towards metal ion sensitivity is evaluated. In DMAEAAm, the tertiary nitrogen atom is a potential site for both proton and metal ion binding, thus conferring on the gel both pH and metal ion sensitivity. The covalently-bound side chains of dimethylamino groups have tertiary nitrogen as the donor atom (Lewis base) and can potentially bind with transition metal ions (Lewis acids). The interaction between dimethylamino side groups and transition metal ions offers high selectivity to its hydrogel towards transition metal ions over non-transition metal ions. For the metal ion sensitivity measurements, gels are first swollen in 0.01 M acetic acid, and then metal ion solution was added successively. The pH of the solution is around 4.0, and at this pH, all dimethylamino ligands are in the protonated state. The measurement of metal ion sensitivity in acetic acid will indicate the ability of dimethylamino ligands present in the hydrogel network to bind the metal ions under more competitive conditions. The weak basic functional groups of the dimethylamino ligand show high affinities towards hydrogen ions as a result metal ion binding in the gel becoming more selective due to the formidable competition from H^+^ under acidic conditions. The addition of 100 µL metal salt solution corresponds to equal molar amounts of metal ions and binding sites.

**Figure 4:**
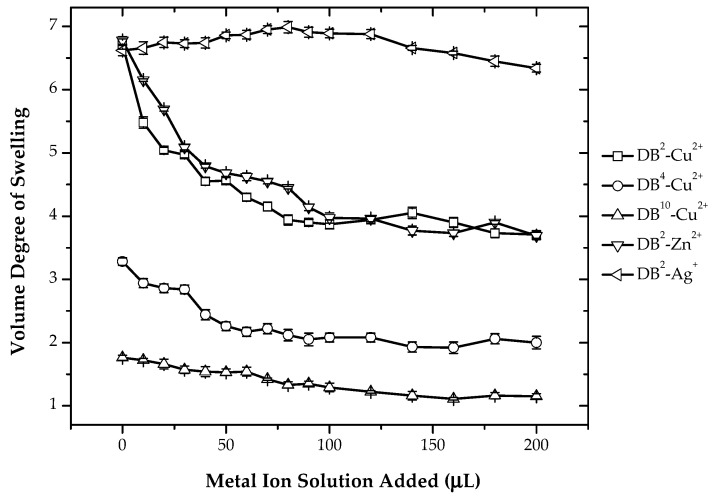
Variation of the volume degree of swelling with respect to varying amounts of CuSO_4_ solution (0.01 M) for different crosslinking densities of hydrogels and variation of the volume degree of swelling of DB^2^ hydrogels with respect to the addition of different metal ions for System I.

Figure 4 shows the variation of the volume degree of swelling with respect to the addition of Cu^2+^ in hydrogels containing different crosslinking density. With the increase in the metal ion concentration, deswelling of hydrogels occurs. This behavior is observed, as in acetic acid medium, most of the dimethylamino ligands are in the protonated state, and hence, the hydrogels are in more hydrophilic state. With the addition of metal ions, the protons are being replaced by metal ions. Thus, hydrogels are in a deprotonated state after the addition of metal ions, which makes the gels less hydrophilic [30]. Therefore, with the successive addition of metal ions, an increasing amount of dimethylamino ligands is deprotonated, and thus, there is deswelling of hydrogels. Further, the metal ions can interact with more than one amino group, resulting in additional crosslinking and, thus, in a deswelling of the gels. The interchain crosslinking is supported by the fact that a rapid decrease in the volume degree of swelling can be observed for the addition of metal salt of 50 µL corresponding to a ratio of functional groups to metal ions of 2:1. Later, the change is less pronounced.

In addition, Figure 4 shows the variation of the volume degree of swelling of DB^2^ hydrogels with respect to the addition of Cu^2+^, Zn^2+^ and Ag^+^. The deswelling behavior was observed with the addition of Cu^2+^ and Zn^2+^, while no change is observed with the addition of Ag^+^ ions. Because of the high affinity of weak basic functionalities towards H^+^ and also because of the lower binding constant or formation constant for Ag+ ions, the protonation is preferred for these ligands. The acid dissociation constants for the tridentate ligand, for example, diethylene triamine [31] are p*K*_a1_ = 4.25, p*K*_a2_ = 8.98 and p*K*_a3_ = 9.70 and for the formation constant of the metal complex (log β) with Cu^2+^ and Zn^2+^ 16.0 and 8.9, respectively. The formation constant (log β) [32] of Ag^+^ with *N,N*-tetramethylethylenediamine is around 3.3–5.9. From these values, it can be inferred that for amine ligands, the formation constant of metal complex is higher as compared to the acid dissociation constant for Cu^2+^ and Zn^2+^. Thus, there is preferential binding of metal ions Cu^2+^ and Zn^2+^ with the dimethylamino ligand. While for Ag^+^ formation, the constant of metal complex is lower as compared to the acid dissociation constant; therefore, there is no preferential binding with Ag^+^ ions.

The interaction of metal ions with the ligand is based on ideas, such as the hard and soft acid and base (HSAB) principle of Pearson [33]. According to this principle, hard bases prefer to bind with hard acids, and soft bases prefer to bind with soft acids and *vice versa*. Based on this principle, the functional groups that are present on the monomer and metal ions that are used in this study can also be classified as hard and soft acids and bases. Thus, in System I, there are dimethylamino groups, which are tertiary amines and hard bases. Cu^2+^ and Zn^2+^ are borderline acids, and Ag^+^ is a soft acid. Therefore, from the HSAB principle, we can expect higher binding of Cu^2+^ and Zn^2+^ to dimethylamino ligands because of the hard acid-hard base interaction as compared to Ag^+^, which is a soft acid and, hence, has lower binding ability with the dimethylamino group.

#### 3.2.2. Effect of the Addition of Acetic Acid on Ionizable Monomer

**Figure 5:**
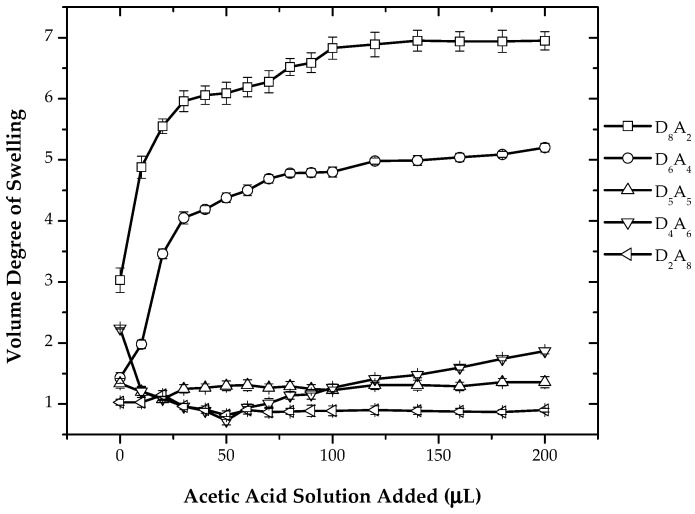
Variation of the volume degree of swelling with the addition of acetic acid for System II.

Due to the relative high ionic strength of the used buffer solutions, the effect of acetic acid addition at low ionic strength was investigated. High ionic strength often screens effects in polyelectrolyte systems. Figure 5 shows the variation of the volume degree of swelling with the successive addition of acetic acid. For the composition having an excess of dimethylamino groups as compared to carboxylic groups, *i.e*., samples D_8_A_2_ and D_6_A_4_, an increase in the volume degree of swelling with respect to the addition of acetic acid was observed. This is due to the protonation of dimethylamino groups, which in turn increases the hydrophilicity and, thus, increases swelling. Samples having an equal amount of acidic and basic groups, *i.e*., sample D_5_A_5_, or samples rich in acidic compound *i.e*., D_4_A_6_ and D_2_A_8_, the swelling behavior showed no change upon the addition of acetic acid. These hydrogels have an excess of weakly-ionizable carboxylic groups. The dimethylamino groups are almost in the protonated state in double-distilled water, and the addition of increasing amounts of acetic acid does not affect the volume degree of swelling.

**Figure 6:**
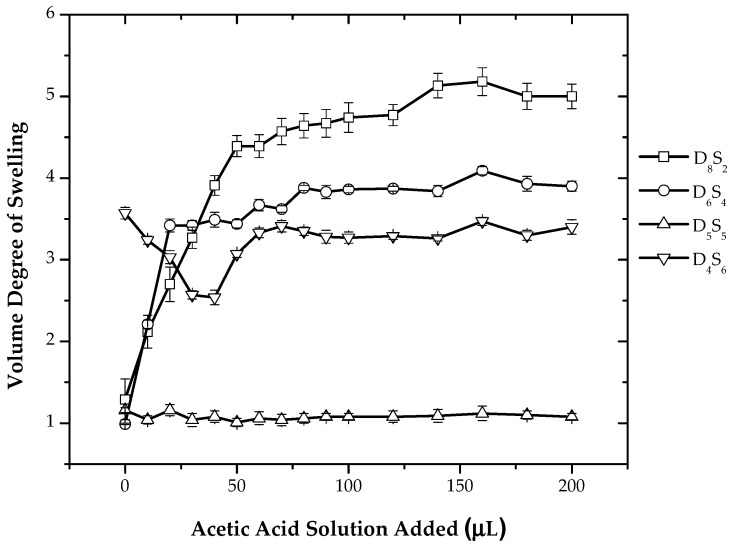
Variation of the volume degree swelling with the addition of acetic acid for System III.

Figure 6 shows the variation of the volume degree of swelling with respect to the addition of acetic acid for System III. For the composition having an excess of dimethylamino ligands, with the addition of acetic acid, an increase in the volume degree of swelling is observed. For the composition having an equal ratio of dimethylamino ligands and sulfonic acid groups or less, there is no change in the volume degree of swelling. This can be explained by the fact that all of the dimethylamino ligands are already in a protonated state due to the ionization of sulfonic acid groups. For the composition having a higher ratio of the sulfonic acid group, the volume degree of swelling is higher as compared to the D_5_S_5_ composition, because of the excess of ionized sulfonic acid groups not involved in ionic crosslinking.

#### 3.2.3. Effect of Ionizable Monomer on Metal Ion Sensitivity

**Figure 7:**
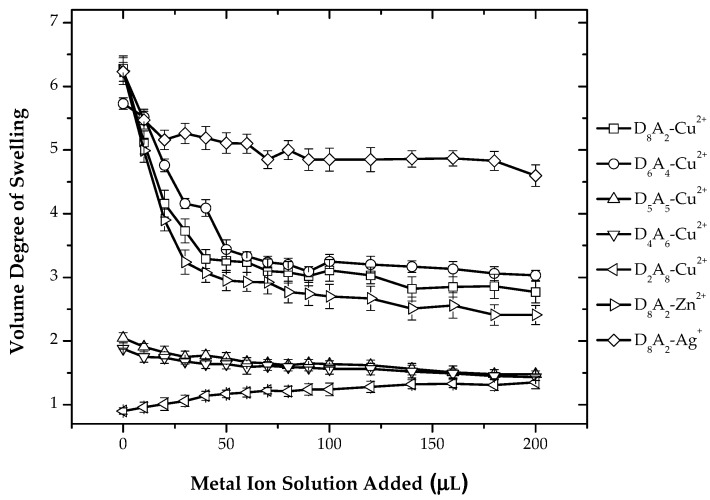
Variation of the volume degree of swelling with respect to varying amounts of CuSO_4_ solution (0.01 M) for all compositions of hydrogels and variation of the volume degree of swelling of D_8_A_2_ hydrogels with respect to the addition of different metal ions for System II.

In Systems II and III, the effect of ionizable monomer on metal ion sensitivity is analyzed. Metal ion measurement in acetic acid shows a decrease in the volume degree of swelling with the addition of Cu^2+^, Zn^2+^, as shown in Figure 7. In the acetic acid medium, most of the carboxylic groups are protonated, but there is still a competition of carboxylic groups being protonated and bound in an ionic complex. Metal ions can bind with a protonated dimethylamino ligand by first deprotonating them, then binding with the ligand. As discussed in System I, binding of metal ions with dimethylamino ligands by the release of protons decreases the hydrophilicity of networks, and thus, deswelling is observed with the successive addition of metal ions. As the carboxylic groups have no role in this case, the deswelling behavior is governed by the dimethylamino group, and thus, as shown in the plots, the degree of deswelling decreased with the decrease in the amount of dimethylamino groups. The addition of Ag^+^ ions again shows no significant change in the volume degree of swelling. The reason for the insignificant decrease in the volume degree of swelling after the addition of AgNO_3_ is discussed above in System I. With increased AA content, the binding capacity of the gels decreased. Gels with no excess of DMEAAm hardly respond to metal salts, because the dimethylamino groups are bound in the ionic complex.

**Figure 8:**
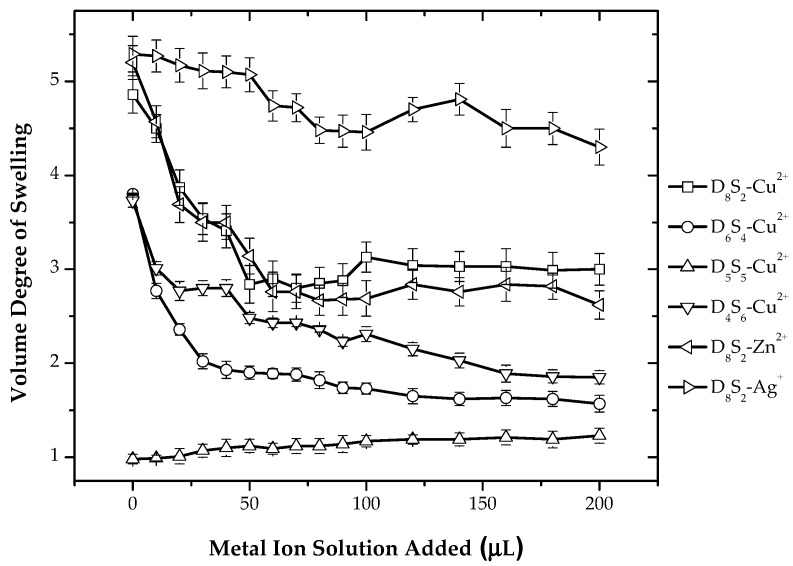
Variation of the volume degree of swelling with respect to varying amounts of CuSO_4_ solution (0.01 M) for all compositions of hydrogels and variation of the volume degree of swelling of D_8_S_2_ hydrogels with respect to the addition of different metal ions for System III.

For System III, metal ion measurement was performed in a similar way as for Systems I and II. In the case of System III, also, there is a decrease in the volume degree of swelling with the successive addition of Cu^2+^ and Zn^2+^, as shown in Figure 8. For the composition having a higher ratio of dimethylamino ligand, *i.e*., D_8_S_2_ and D_6_S_4_, with the addition of metal ions, there is a decrease in the volume degree of swelling. The replacement of protons with metal ions causes a decrease in hydrophilicity and, in turn, decreases the volume degree of swelling. For the hydrogels having composition D_5_S_5_, *i.e*., having an equal ratio of dimethylamino and sulfonate groups, there is no change in the volume degree of swelling at all. The possible explanation for this is that metal ions are not able to bind dimethylamino ligands by first breaking ionic interactions. For hydrogel D_4_S_6_ having a higher amount of sulfonic acid groups with the addition of metal ions, there is deswelling observed; the possible explanation for this is neutralization of the charges of sulfonic acid groups by metal ions. The addition of Ag^+^ showed no change in the volume degree of swelling due to the reasons explained for Systems I and II.

Based on these results, it can be concluded that the DMAEAAm moiety shows sensitivity to Cu^2+^ and Zn^2+^ ions under acidic conditions. If DMAEAAm moieties are present in excess in any system, deswelling of hydrogels is observed with the successive addition of selective metal ions. The reasons for deswelling as discussed above are the deprotonation of DMEAAm moieties by metal ions, as well as metal ions interacting with more than one amino group, resulting in additional crosslinking. In the case of hydrogels containing the weakly-acidic component (AA) in addition to DMAEAAm, sensitivity to metal ions is mostly controlled by the binding of metal ions to the DMAEAAm moiety. Weakly acidic components are not able to bind to metal ions, as they are in the protonated state. For systems containing a component that is in an ionized state in all pH conditions (AMPS) in addition to DMAEAAm, deswelling is observed both due to the binding of metal ions to dimethylamino, as well as the sulfonate component.

## 4. Conclusions

In this study, pH- and metal ion-sensitive hydrogels were synthesized, and their swelling behavior under different pH conditions and with different metal ions was investigated. The introduction of multi-sensitive components into hydrogels can enhance the potential of such materials for fabricating sensors. The introduction of monomer *N*-[2-(dimethylaminoethyl)acrylamide] (DMAEAAm) confers both pH and metal ion sensitivity on the hydrogels. Introduction of other ionizable monomers, like acrylic acid (AA, weakly ionizable) and 2-acrylamido-2-methyl-1-propanesulphonic acid (AMPS, highly ionizable), results in polyampholyte networks. Variation of the crosslinking density and the ratio of the composition of ionizable monomers generated different responses of the volume degree of swelling. Thus, the desired response can be obtained by fine-tuning the ratio of ionizable components or by varying crosslinking density. The preliminary pH sensitivity measurement establishes the different pH ranges in which hydrogels have different characteristics due to the presence of additional interactions, which develop as the result of the presence of different species in the network structure. Metal ion sensitivity measurements were done under acidic conditions to understand the sensitivity of these hydrogels towards metal ions, like Cu^2+^, Zn^2+^ and Ag^+^, in a competitive environment. It was observed that different responses were obtained under different conditions of measurement and different pre-treatment conditions. The response of hydrogels towards the addition of metal ions depends largely on the initial state of existence of the hydrogel network, which in-turn depends on various factors, like the composition of hydrogels and the pH of the external solution. Thus, the desired response can be obtained by adjusting the parameters, like the composition, pH and initial state of the hydrogels. It can be concluded that this study provides detailed information on the synthesis and characterization of the swelling behavior of pH- and metal ion-sensitive hydrogels under different pH conditions, with different types of metal ions and varying concentrations of metal ions.

## Figures and Tables

**Table 1:** Composition of hydrogels prepared for Systems I, II and III. **D**, *N*-[2-(dimethylaminoethyl)acrylamide]; **M**, *N*,*N*-dimethylacrylamide; **B**, *N,N*-methylenebis¬acrylamide; **A**, acrylic acid, **S**, 2-acrylamido-2-methyl-1-propanesulphonic acid [(Irgacure 2959, 0.1 mmol; distilled water, 3.5 mL). The superscript **a** stands for the concentration of crosslinker in % (mmol/mmol) with respect to the monomer concentration; b and c stand for the concentration of monomers **D** and **A** in mmol multiplied by four; **d** and **e** stand for the concentration of monomers **D** and **S** in mmol multiplied by four.

**System I**				
Designation used **DB ^a^**	DMAEAAm (**D**) (mmol)		DMAAm (**M**) (mmol)	BIS (**B**) (mmol)
**DB ^1^**	2.5		2.5	0.05
**DB ^2^**	2.5		2.5	0.1
**DB ^4^**	2.5		2.5	0.2
**DB ^10^**	2.5		2.5	0.5
**System II**				
Designation used **D_b_A_c_**	DMAEAAm (**D**) (mmol)	AA (**A**) (mmol)	DMAAm (**M**) (mmol)	BIS (**B**) (mmol)
**D_8_A_2_**	2.0	0.5	2.5	0.1
**D_6_A_4_**	1.5	1.0	2.5	0.1
**D_5_A_5_**	1.25	1.25	2.5	0.1
**D_4_A_6_**	1.0	1.5	2.5	0.1
**D_2_A_8_**	0.5	2.0	2.5	0.1
**System III**				
Designation used **D_d_S_e_**	DMAEAAm (**D**) (mmol)	AMPS (**S**) (mmol)	DMAAm (**M**) (mmol)	BIS (**B**) (mmol)
**D_8_S_2_**	2.0	0.5	2.5	0.1
**D_6_S_4_**	1.5	1.0	2.5	0.1
**D_5_S_5_**	1.25	1.25	2.5	0.1
**D_4_S_6_**	1.0	1.5	2.5	0.1
**D_2_S_8_**	0.5	2.0	2.5	0.1

## Abbreviations

The following abbreviations are used in this manuscript:

DMAEAAm: *N*-[2-(dimethylaminoethyl)acrylamide]
DMAAm: *N,N*-dimethylacrylamide
AA: Acrylic acid
AMPS: 2-acrylamido-2-methyl-1-propanesulphonic acid
BIS: *N,N*-Methylenebisacrylamide
PBS: Phosphate-buffered saline
Irgacure 2959: 2-Hydroxy-1-[4-(2-hydroxyethoxy) phenyl]-2-methyl-1-propanone

## References

[1] Beebe D.J., Moore J.S., Bauer J.M., Yu Q., Liu R.H., Devadoss C., Jo B.H. (2000). Functional hydrogel structures for autonomous flow control inside microfluidic channels. Nature.

[2] Eddington D.T., Liu R.H., Moore J.S., Beebe D.J. (2001). An organic self-regulating microfluidic system. Lab. Chip.

[3] Ionov L. (2014). Hydrogel-based actuators: possibilities and limitations. Mater. Today.

[4] Döring A., Birnbaum W., Kuckling D. (2013). Responsive hydrogels – structurally and dimensionally optimized frameworks for application in catalysis, micro-system, technology and material science. Chem. Soc. Rev..

[5] Katchalsky A., Michaeli I. (1955). Polyelectrolyte gels in salt solutions. J. Polym. Sci..

[6] Brannon-Peppas L., Peppas N.A. (1991). Equilibrium swelling behavior of pH-sensitive hydrogels. Chem. Eng. Sci..

[7] Oppermann W. (1992). Polyelectrolyte gels. Proceedings of the ACS Symposium Series No. 480.

[8] Siegel R.A., Firestone B.A. (1988). pH-dependent equilibrium swelling properties of hydrophobic polyelectrolyte copolymer gels. Macromolecules.

[9] Cornejo-Bravo J.M., Siegel R.A. (1996). Water vapour sorption behaviour of copolymers of *N*,*N*-diethylaminoethyl methacrylate and methy methacrylate. Biomaterials.

[10] Bashir R., Hilt J.Z., Elibol O., Gupta A., Peppas N.A. (2002). Micromechanical cantilever as an ultrasensitive pH microsensor. Appl. Phy. Lett..

[11] Liu R.H., Yu Q., Beebe D.J. (2002). Fabrication and characterization of hydrogel-based microvalves. J. Microelectromech. Syst..

[12] Yajima Y., Yamada M., Yamada E., Iwase M., Seki M. (2014). Facile fabrication processes for hydrogel-based microfluidic devices made of natural biopolymers. Biomicrofluidics.

[13] Tanaka T. (1978). Collapse of gels and the critical endpoint. Phys. Rev. Lett..

[14] Hirotsu S., Hirokawa Y., Tanaka T. (1987). Volume phase transition of ionized *N*-isopropylacrylamide gels. J. Chem. Phys..

[15] Beltran S., Baker J.P., Hooper H.H., Blanch H.W., Prausnitz J.M. (1991). Swelling equilibria for weakly ionizable, temperature-sensitive hydrogels. Macromolecules.

[16] Taylor L.D., Ceranknowski L.D. (1975). Preparation of films exhibiting a balanced temperature dependence to permeation by aqueous solution–A study of lower consolute behavior. J. Polym. Sci. Part A.

[17] Prochazka K., Matin T.J., Webber S.E., Munk P. (1996). Onion-type micelles in aqueous media. Macromolecules.

[18] Kuckling D., Adler H.-J.P., Arndt K.F., Ling L., Habicher W.D. (2000). Temperature and pH dependent solubility of novel poly(*N*-isopropylacrylamide)-copolymers. Macromol. Chem. Phys..

[19] Wang X., Ye G., Wang X. (2014). Hydrogel diffraction gratings functionalized with crown ether for heavy metal ion detection. Sens. Actuators B.

[20] Pi S.-W., Ju X.-J., Wu H.-G., Xie R., Chu L.-Y. (2010). Smart responsive microcapsules capable of recognizing heavy metal ions. J. Colloid Interface Sci..

[21] Luo Q., Guan Y., Zhang Y., Siddiq M. (2010). Lead-sensitive PNIPAM microgels modified with crown ether groups. J. Polym. Sci. Part A.

[22] Kuckling D., Pareek P. (2003). Synthesis of transition-metal-ion-selective poly(*N*-isopropylacrylamide) hydrogels by the incorporation of an aza crown ether. J. Polym. Sci..

[23] Mayes A.G., Blyth J., Millington R.B., Lowe C.R. (2002). Metal ion-sensitive holographic sensors. Anal. Chem..

[24] Holtz J.H., Hotlz J.S., Munro C.H., Asher S.A. (1998). Intelligent polymerized crystalline colloidal arrays: novel chemical sensor materials. Anal. Chem..

[25] Holtz J.H., Asher S.A. (1997). Polymerized colloidal crystal hydrogel films as intelligent chemical sensing material. Nature.

[26] Bünemann H., Dattagupta N., Schuetz H.J., Müller W. (1981). Synthesis and properties of acrylamide-substituted base pair specific dyes for deoxyribonucleic acid template mediated synthesis of dye polymers. Biochemistry.

[27] Gao M., Gawel K., Stokke B.T. (2014). Polyelectrolyte and antipolyelectrolyte effects in swelling of polyampholyte and polyzwitterionic charge balanced and charge offset hydrogels. Eur. Polym. J..

[28] Ihsan A.B., Sun T.L., Kuroda S., Haque M.A., Kurokawa T., Nakajima T., Gong J.P. (2013). A phase diagram of neutral polyampholyte—From solution to tough hydrogel. J. Mater. Chem. B.

[29] Masaki M., Kokufuta E. (2013). Polyampholyte gels of a cross-linked polyanion or polycation network into which an oppositely charged polyion was immobilized. Colloid Polym. Sci..

[30] Cai Q.Y., Grimes C.A. (2000). A remote query magnetoelastic pH sensor. Sens. Actuators B.

[31] Yang R., Zompa L.J. (1976). Metal complexes of cyclic triamines. 1. Complexes of 1,4,5-triazacyclononane ([9]aneN3) with nickel (II), copper (II), and zinc (II). Inorg. Chem..

[32] Hilliard H.M., Yoke J.T. (1966). Silver(I) complexes of bicyclic tertiary amines. Inorg. Chem..

[33] Pearson R.G. (1963). Hard and soft acids and bases. J. Am. Chem. Soc..

